# Optimal management of adults with pharyngitis – a multi-criteria decision analysis

**DOI:** 10.1186/1472-6947-6-14

**Published:** 2006-03-13

**Authors:** Sonal Singh, James G Dolan, Robert M Centor

**Affiliations:** 1 Department of Medicine, Wake Forest University, Winston Salem, NC, USA.; 2 Department of Medicine, Unity Health System and the University of Rochester, Rochester, New York, USA.; 3 Department of Medicine, University of Alabama-Birmingham, Birmingham, AL, USA.

## Abstract

**Background:**

Current practice guidelines offer different management recommendations for adults presenting with a sore throat. The key issue is the extent to which the clinical likelihood of a Group A streptococcal infection should affect patient management decisions. To help resolve this issue, we conducted a multi-criteria decision analysis using the Analytic Hierarchy Process.

**Methods:**

We defined optimal patient management using four criteria: 1) reduce symptom duration; 2) prevent infectious complications, local and systemic; 3) minimize antibiotic side effects, minor and anaphylaxis; and 4) achieve prudent use of antibiotics, avoiding both over-use and under-use. In our baseline analysis we assumed that all criteria and sub-criteria were equally important except minimizing anaphylactic side effects, which was judged very strongly more important than minimizing minor side effects. Management strategies included: a) No test, No treatment; b) Perform a rapid strep test and treat if positive; c) Perform a throat culture and treat if positive; d) Perform a rapid strep test and treat if positive; if negative obtain a throat culture and treat if positive; and e) treat without further tests. We defined four scenarios based on the likelihood of group A streptococcal infection using the Centor score, a well-validated clinical index. Published data were used to estimate the likelihoods of clinical outcomes and the test operating characteristics of the rapid strep test and throat culture for identifying group A streptococcal infections.

**Results:**

Using the baseline assumptions, no testing and no treatment is preferred for patients with Centor scores of 1; two strategies – culture and treat if positive and rapid strep with culture of negative results – are equally preferable for patients with Centor scores of 2; and rapid strep with culture of negative results is the best management strategy for patients with Centor scores 3 or 4. These results are sensitive to the priorities assigned to the decision criteria, especially avoiding over-use versus under-use of antibiotics, and the population prevalence of Group A streptococcal pharyngitis.

**Conclusion:**

The optimal clinical management of adults with sore throat depends on both the clinical probability of a group A streptococcal infection and clinical judgments that incorporate individual patient and practice circumstances.

## Background

Sore throat is one of the most common illnesses for which patients consult their physicians in the United States with an estimated 6.7 million adult visits to primary care providers each year; nearly three-quarters of these visits result in antibiotic prescriptions [1,2]. The Group A streptococcus is the most common bacterial cause of acute pharyngitis, accounting for 5–26% cases in adults. Higher rates occur in emergency room and urgent care settings and during the winter and early spring [3-5].

A well-validated clinical algorithm for estimating the likelihood of a Group A streptococcal infection in a patient presenting with a sore throat called the Centor Score is available [6]. The Centor score is calculated by determining how many of the following four clinical features are present: history of fever, tonsillar exudates, anterior cervical adenopathy, and absence of cough. In a variety of adult populations, the prevalence of Group A streptococcal infections has been shown to be proportional to the Centor score. For example, when the population prevalence is 10%, the likelihood of a Group A streptococcal infection ranges from 3% if one feature is present to 41% when all four exist [3].

Despite the existence of this clinical prediction rule and extensive medical literature on this topic, conflicting approaches to the diagnosis and management of sore-throat are currently proposed in guidelines issued by the Infectious Diseases Society of America (IDSA) on one hand [7], and American College of Physicians (ACP), in collaboration with the Centers for Disease Control and the American Academy of Family Physicians [8,9], on the other. Both guidelines identify the major issue in sore throat management as the identification of patients with Group A streptococcal infections for whom antibiotic treatment is indicated, but they differ in the usefulness they ascribe to the clinical estimation of the likelihood Group A streptococcal infection in making management decisions. Both guidelines agree that no testing or treatment is appropriate for patients with a low likelihood of Group A Streptococcal pharyngitis, based on a Centor score of 1. The differences occur in management recommendations for patients with Centor scores 2 and above. The IDSA recommends laboratory confirmation of a Group A streptococcal infection in all cases whereas the ACP guideline endorses several management options, including empiric treatment, for patients with Centor scores of 3 and 4.

To help clinicians better understand the differences between these two conflicting guidelines and decide which one they should follow, we conducted a multi-criteria decision analysis using the Analytic Hierarchy Process (AHP).

## Methods

The Analytic Hierarchy Process (AHP) is one of several multi-criteria decision analysis methods designed to help people make better decisions in complex situations involving tradeoffs between the advantages and disadvantages of several alternatives [10]. An AHP analysis can be divided into five steps.

The first step consists of defining the goal of the decision, the alternatives being considered, and the criteria that will be used to determine how well the alternatives can be expected to meet the goal. In addition, different decision perspectives and/or scenarios can be defined. These decision elements are then organized into a hierarchical decision model with the goal at the top, the alternatives at the bottom, and the criteria in between. The model serves as both a description of the decision problem and a framework for dividing the decision into smaller, more manageable components for subsequent analysis.

In the second step of an AHP analysis, information about how well the alternatives can be expected to fulfil the decision criteria is gathered and summarized.

The third step consists of comparing the alternatives' abilities to fulfil the criteria and judging the importance of the criteria relative to the goal of the decision. If the model includes different decision perspectives or scenarios, separate judgements are made for each. The recommended format for making all of these judgements is a series of pairwise comparisons between two elements at a time. After all the comparisons are made they are combined to create a normalized, ratio scale that summarizes the results of the direct and indirect comparisons made among the decision elements. The internal consistency of the judgements within a set of pairwise comparisons is routinely assessed using a measure called the consistency ratio. A consistency ratio of 0 indicates perfect consistency. By convention, consistency ratios less than 0.1 are considered acceptable.

In the fourth step of the AHP, the scales created in step 3 are combined to create a summary score indicating how well the alternatives can be expected to meet the goal. This is done in a manner that is analogous to calculating a weighted average by multiplying the scores indicating how well alternatives fulfil the criteria by the priorities assigned to the criteria and adding the results. The resulting scores, which add up to one and are commonly expressed as percentages, indicate the alternatives' relative abilities to fulfil the goal of the decision.

The fifth step consists of sensitivity analyses to explore the effects of changing the data or judgements used in the original analysis.

These steps, as they were carried out in the conduct of this analysis, are illustrated below. More detailed descriptions of the AHP have been published previously [11-13].

### Sore throat management analysis step one

We performed the analysis from the perspective of a primary care practitioner in an office setting seeing an adult patient aged 18 years or older with a chief complaint of sore throat. The goal was to identify optimal office management of such a patient. We defined four criteria for determining optimal management: 1) To shorten the duration of illness; 2) To prevent infectious complications; 3) To minimize antibiotic side effects; and 4) Optimal antibiotic use. The criterion preventing infectious complications was broken down into two sub-criteria: prevent local complications (peri-tonsillar abscess) and prevent systemic complications (acute rheumatic fever). Avoid antibiotic side effects was divided into minor (rash, gastrointestinal distress) and major (anaphylaxis). Optimal antibiotic use was divided into avoiding under-treatment with antibiotics to reduce the chance of potentially preventable spread of disease to household and other contacts and avoiding over-treatment to reduce unnecessary antibiotic use and the development of bacterial antibiotic resistance.

We examined five potential management strategies:

• No testing and no treatment (NO TEST, NO TREAT);

• Obtain a rapid streptococcal antigen test and treat patients who test positive, do not treat patients who test negative (RAPID STREP);

• Obtain a throat culture and treat patients who test positive, do not treat if negative (CULTURE);

• Obtain a rapid streptococcal antigen test and treat patients who test positive. Obtain a throat culture on patients who test negative and treat if the culture is positive (RAPID STREP & CULTURE);

• Treat everyone with no further diagnostic testing. (EMPIRIC TREATMENT).

**Figure 1:**
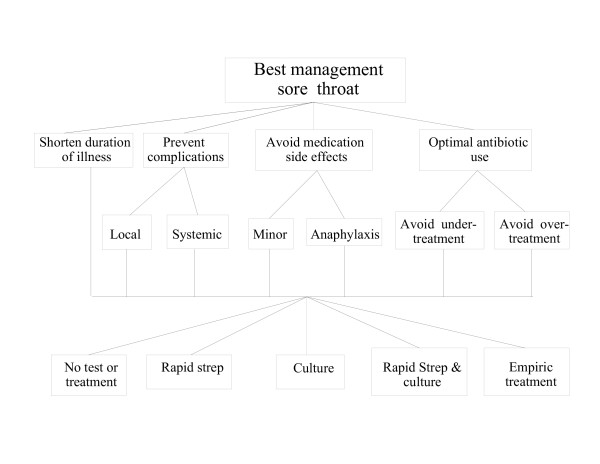
The decision model. Please refer to the text for definitions of the decision criteria and sub-criteria on middle two levels and the management alternatives on the bottom level.

To examine the effects of different likelihoods of Group A streptococcal pharyngitis, we created four clinical scenarios, one for each of the four possible Centor score results. To make our analysis comparable with earlier studies, we assumed a population prevalence of Group A streptococcal pharyngitis of 10% and, based on published data in adult patients coming to the primary care office in the US, used the following estimates for the prevalence of Group A Streptococcal pharyngitis in each scenario: 3% for Centor score 1, 8% for Centor score 2, 19% for Centor score 3, and 41% for Centor score 4 [3,5]. The resulting decision model is shown in Figure 1. The effects of increasing the population prevalence to 20% were explored in a sensitivity analysis, described below.

### Step two – assemble and organize outcome information

To simplify the analysis, we limited it to patients who are not known to be allergic to penicillin. We assumed that all untreated episodes of pharyngitis, bacterial or non-bacterial, produce five days of symptoms [14] and that patients are being seen within three days of symptom onset. We also assumed that all treated patients would receive a 10-day course of oral penicillin therapy and that penicillin provides two days of symptom improvement if begun immediately and one day of improvement if delayed 24 hours while awaiting culture results [15]. We estimated the mean duration of symptoms for each management option using Bayes's theorem, the prevalence of Group A streptococcal infections, and the test operating characteristics of rapid streptococcal antigen tests and throat cultures to calculate the number of patients in each scenario with true and false positive and negative test results. The data used to perform these calculations are summarized in Table 1[5,16]. We then determined the number of patients per thousand in each scenario with Group A streptococcal pharyngitis who would be treated and reduced their symptom duration based on when their treatment would begin under each management option. The total number of symptomatic days for the entire cohort was then calculated and divided by 1,000 to derive the mean duration of illness.

The likelihoods of the other outcomes were estimated in a similar way. The risks of local and systemic complications were based on the prevalence of patients in each option with treated and untreated Group A streptococcal infections and the estimated likelihoods of complications in each group. We calculated the chances of antibiotic side effects by multiplying the proportion of patients in each management group who would be treated with penicillin times their estimated risk of side effects [5,15,17]. The likelihood of over-treatment with antibiotics associated with each management strategy was determined by calculating the likelihood that patients without Group A streptococcal infections would be treated. We determined under-treatment with antibiotics by calculating the likelihood that patients with Group A streptococcal infections would not be treated under each management strategy.

The resulting outcome estimates associated with the alternative management strategies for each clinical scenario are summarized in Additional file 1 [see Additional file 1]. To facilitate comparisons among the outcomes, similar results were grouped into categories that are also included in Additional file 1 [see Additional file 1].

### Step three – make comparisons

#### a) Comparisons among alternatives relative to the criteria

We compared the management strategies' abilities to shorten the duration of illness by dividing the reciprocals of the mean duration of illness associated with each strategy (since a shorter duration of illness is preferable).

Comparisons among the alternatives with regard to the other criteria were made using standard AHP pairwise comparisons among the outcome categories defined in the previous step. Because of the large number of outcomes that are possible if all four Centor groups are combined, these comparisons were made separately for each Centor group. Detailed information about these comparisons is included in Additional file 2 [see Additional file 2].

#### b) Comparisons among the criteria

In the baseline analysis we assumed that all criteria and sub-criteria are equally important in making a management decision with one exception: in terms of avoiding penicillin side effects, we judged avoiding an anaphylactic reaction very strongly more important than avoiding a minor reaction such as a skin rash. We explored how different judgments regarding the priorities of the criteria affected the results in a series of sensitivity analyses, further described below.

### Step four – combine all judgments to determine how well alternatives can be expected to meet the goal

We used the standard AHP weighted averaging method to combine the results of the judgments made in step three to determine the relative abilities of the management options to meet the goal for each of the four clinical scenarios. We defined relative differences between options of ≥ 1.1 as significant.

### Step five – sensitivity analysis

We explored the impact of different judgments regarding the relative importance of the criteria and sub-criteria by varying their priorities from 0 (of no importance) to 1 (the only consideration that is important) and recalculating the alternatives' overall scores. We also repeated the baseline analysis assuming that the prevalence of Group A streptococcal infections was 20%. The data used for this analysis are summarized in Additional file 3 [see Additional file 3]. Details about the pairwise comparisons among the outcome estimates used for this analysis are contained in Additional file 4 [see Additional file 4].

All AHP analyses were conducting using *Expert Choice 2000*, a standard AHP software program [18].

## Results

### Baseline analysis

**Figure 2:**
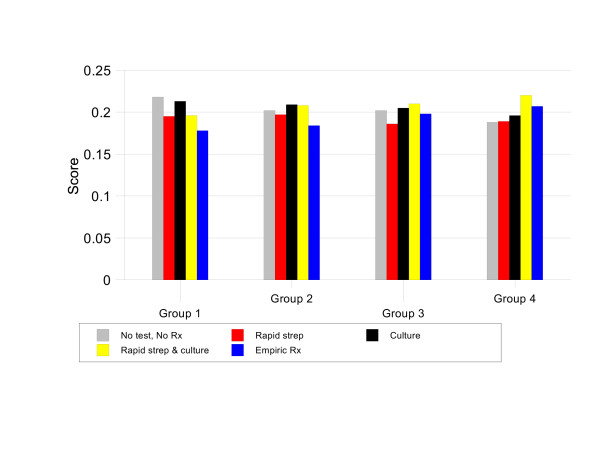
Results of baseline analysis.

The results of the baseline analysis are summarized in Figure 2. The most preferred strategies are NO TEST, NO TREAT for patients with a Centor score of 1, CULTURE or RAPID STREP & CULTURE for patients with a Centor score of 2, and RAPID STREP & CULTURE for patients with Centor scores of 3 or 4.

**Figure 3:**
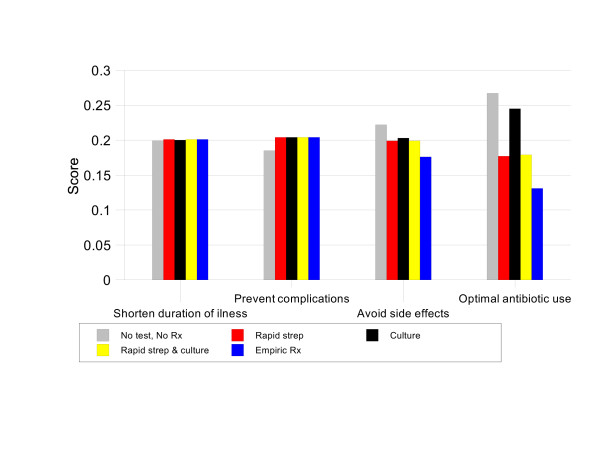
Results by criteria, Group 1 baseline analysis.

For patients with Centor scores of 1, NO TEST, NO TREAT has the highest priority score, 21.8%. This score is 1.02 times better than CULTURE (priority score 21.3%), 1.11 times better than RAPID STREP & CULTURE, 1.12 times better than RAPID STREP, and 1.22 times better than EMPIRIC TREATMENT. Figure 3 illustrates the strengths and weaknesses of the management strategies in this situation. NO TEST, NO TREAT is the best option in terms of avoiding antibiotic side effects and optimal antibiotic use. These advantages more than compensate for its relatively weak performance in preventing infectious complications.

**Figure 4:**
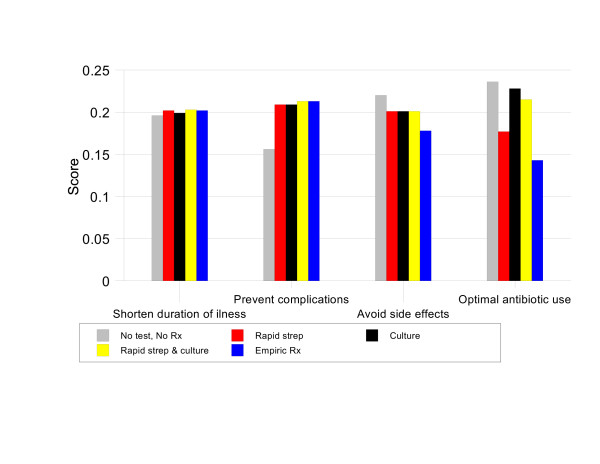
Results by criteria, Group 2 baseline analysis.

In patients with a Centor score of 2, CULTURE and RAPID STREP & CULTURE are tied for most preferred strategy with priority scores of 20.9% and 20.8% respectively. These two options are 1.03 times better than NO TEST NO TREAT (priority score 20.2%), 1.06 times better than RAPID STREP (priority score 19.7%), and 1.14 times better than EMPIRIC TREATMENT (priority score 18.4%). Figure 4 shows that this is because these two strategies are substantially better than EMPIRIC TREATMENT and RAPID STREP in terms of optimal antibiotic use and better than NO TEST, NO TREAT in preventing complications.

**Figure 5:**
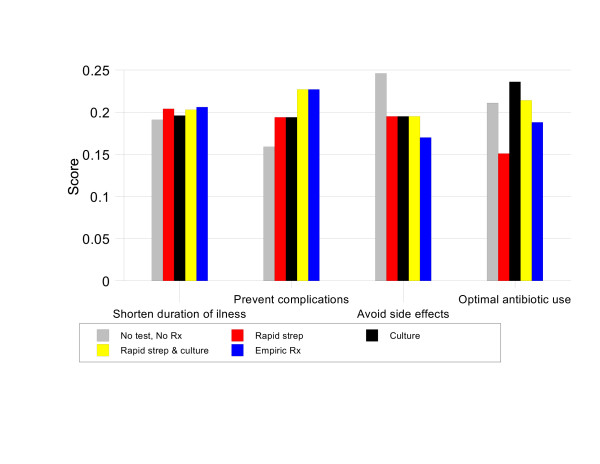
Results by criteria, Group 3 baseline analysis.

For patients with a Centor score of 3, the preferred strategy is RAPID STREP & CULTURE, priority score 21%. This option is 1.02 times better than CULTURE (priority score 20.5%), 1.04 times better than NO TEST, NO TREAT (priority score 20.2%), 1.06 times better than EMPIRIC TREATMENT (priority score 19.8%), and 1.13 times better than RAPID STREP (priority score 18.6%). Figure 5 illustrates that, in this group, RAPID STREP & CULTURE is the best strategy for preventing complications, optimizing antibiotic use, and shortening the duration of symptoms.

**Figure 6:**
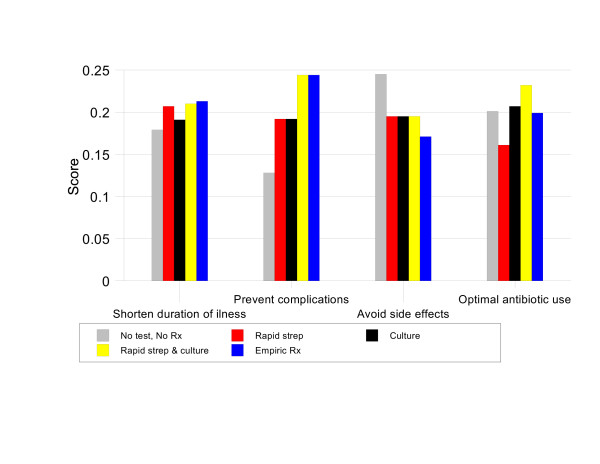
Results by criteria, Group 4 baseline analysis.

For patients with a Centor score of 4, RAPID STREP & CULTURE is again the best management strategy with a priority score 22.0%. It is 1.06 times better than EMPIRIC TREATMENT (priority score 20.7%), 1.12 times better than CULTURE (priority score 19.6%), 1.16 times better than RAPID STREP (priority score 18.9%) and 1.17 times better than NO TEST, NO TREAT (priority score 18.8%). As shown in Figure 6, the main advantage of RAPID STREP & CULTURE in this situation is its ability to both prevent complications and promote optimal antibiotic use.

### Sensitivity analyses

In the decision model, there are 28 possible changes in the relative priorities assigned to the decision criteria and sub-criteria. The sensitivity analysis revealed that 14 of these changes resulted in a change in preferred management strategy from the one(s) identified using the baseline assumptions. These results are summarized in Table 2. Changes in preferred management strategy were associated with alterations in the priorities of all criteria except preventing local versus systemic infectious complications.

**Figure 7:**
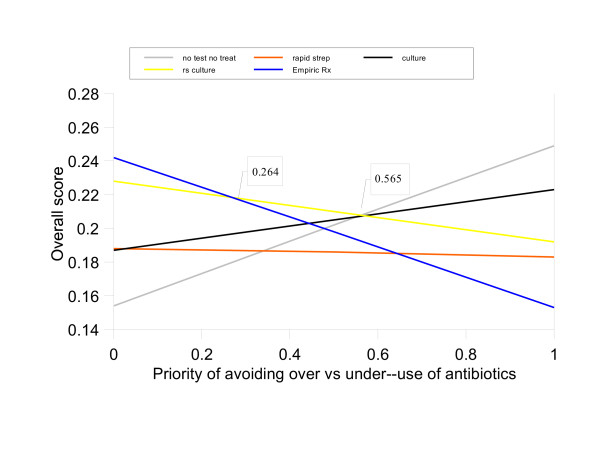
One-way sensitivity analysis, priorities of avoid over-use versus under-use of antibiotics for Centor Group 3 patients.

The most changes were associated with differences in the relative priorities of avoiding over-use versus under-use of antibiotics, which was the only criterion that affected the results of all four Centor groups. These results are illustrated in Figure 7, which shows the effects of varying the priorities assigned to these two sub-criteria relative to their parent criterion, Optimal antibiotic use, for Centor group 3. EMPIRIC TREATMENT is the most preferred management strategy when the priority of avoid over-use of antibiotics is between 0 and 26.4%. RAPID STREP & CULTURE is preferred when the priority of avoid over-use is between 26.5% and 56.5%; this interval includes the baseline value of 50%. For higher priorities, NO TEST, NO TREAT is preferred. Similar "double-thresholds" also occurred in Centor groups 2 and 4. Additional graphs illustrating these one-way sensitivity analysis results are included in Additional file 5 [see Additional file 5].

**Figure 8:**
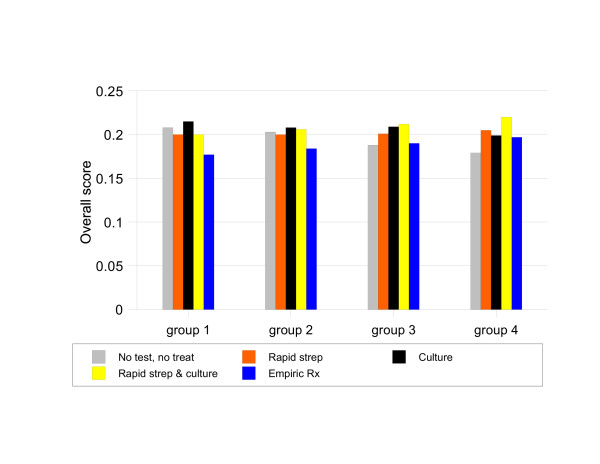
Sensitivity analysis, 20% population prevalence of Group A streptococcal pharyngitis.

The results of increasing the population prevalence of Group A streptococcal pharyngitis from 10% to 20% in the baseline analysis are illustrated in Figure 8. CULTURE becomes the preferred strategy for patients with Centor scores of 1 or 2 whereas RAPID STREP & CULTURE remains the preferred strategy for patients with Centor scores 3 or 4.

## Discussion

Because of its frequency, optimal management of patients presenting with a sore throat is an important quality of care issue in ambulatory settings. The best way to manage an adult patient with a sore throat, however, has been hard to determine. Different management strategies have been advocated, even within the same institution [19]. Ironically, the uncertainty about appropriate management was heightened, not lessened, by the recent publication of two clinical guidelines that make different patient management recommendations [7,9].

The results of our analysis help explain why it is so difficult to identify the best way to manage this common clinical problem and provide guidance for clinicians seeking to identify the optimal approach to managing adult patients with sore throats. As illustrated by the baseline analysis, there is no clearly superior patient management strategy. In almost every scenario, as illustrated by the small differences in priority scores, there are at least two or three management strategies that are very similar in their abilities to meet all of the decision criteria. The sensitivity analyses show that the choice of management strategy depends on the relative priorities assigned to the criteria being used to evaluate the alternatives and the clinical likelihood of Group A streptococcal pharyngitis. The most important judgment among the criteria is the relative priority of avoiding over-use of antibiotics, which is important to reduce the development of bacterial resistance to antibiotics, versus avoiding under-use, which is important to prevent the spread of infection to household members and other close contacts. Although mentioned as a consideration in both the IDSA and ACP guidelines, neither identifies reducing the spread of infection as a major factor in making treatment decisions. This result suggests that the importance of reducing the spread of infection in making treatment decisions may have been underestimated in both sets of guidelines.

These findings suggest that differences in decision criteria and/or the priorities assigned to them account for some, if not all, of the discrepancies in current recommendations regarding the management of adult patients with a sore throat. They further suggest that it may not be possible to define a universally preferred management strategy, making this clinical problem unsuitable for traditionally formulated clinical guidelines. Rather, it may be necessary to develop and implement a flexible guideline approach that combines current clinical evidence, including the population prevalence of Group A streptococcal pharyngitis, with the preferences of the individuals involved in clinical management decisions [20].

The results of our analysis are consistent with those of McIsaac and colleagues who compared the management strategies proposed in the same two guidelines in a population of patients with sore throats presenting with a Centor score of 2 or more who had both rapid strep tests and throat cultures performed [21]. Using throat culture as the reference standard for diagnosing a group A streptococcal infection, they found that the best management strategies were to either culture everyone and only treat patients with positive results or to culture patients with Centor scores of 2 or 3 and empirically treat those with a score of 4. They concluded, however, that the optimal management of patients with sore throat depends on trade-offs among the priorities assigned to multiple decision criteria including identifying a group A streptococcal infection, avoiding over-use of antibiotics, the burden of office-based testing, the value of immediate test results, and the need for telephone follow-up of throat culture results.

Our results are also compatible with those of Neuner and colleagues who compared the same management strategies using cost-effectiveness analysis [5]. They found that, unless the prevalence of group A streptococcal infections is greater than 70%, all strategies except empiric treatment were reasonable and cost-effective.

Our study differs from these two previous ones in several ways. First, both assumed that the throat culture is 100% sensitive and specific. Although the throat culture is used as a diagnostic standard in practice, there is good evidence that it is not a perfect diagnostic test for group A streptococcal pharyngitis in clinical settings due to variations in culture techniques and the presence of asymptomatic carriers [16]. For this reason we used lower values for the sensitivity and specificity of the throat culture to more closely represent the operating characteristics of this test in routine practice settings.

The other major difference is that multi-criteria method of analysis we used provides a way to examine the how trade-offs among different management objectives affect the choice of management strategy that is not possible with standard methods of cost-effectiveness analysis.

This analysis has several limitations. It was limited to patients in office settings who can be treated with penicillin and it is possible that the results could be different if other treatment settings or regimens were considered. We also used literature-derived estimates for the sensitivity and specificity of the rapid strep tests and throat cultures, the effect of antibiotic treatment on duration of illness, and the prevalence of Group A streptococcal infections that could be inaccurate. A third limitation is that, in keeping with current guidelines, we assumed that non-group A streptococcal organisms do not merit antibiotic treatment. There is evidence, however, that patient outcomes may be improved if non-group A streptococcal organisms, especially group C, are treated [22]. We also did not specifically include the considerations about office efficiency and the value of immediate microbiologic results that were identified by McIsaac and colleagues as potentially important factors in determining patient management in busy office settings [21]. Finally, because we chose to compare the outcomes of each clinical scenario separately, it is not possible to directly compare the priority scores across the different scenarios.

## Conclusion

In summary, we found that optimal management of adult patients presenting with a sore throat depends on both the clinical likelihood of a group A streptococcal infection and the relative importance assigned to the criteria used to define good patient management, especially avoiding over-use versus under-use of antibiotics and preventing infectious complications. These findings suggest that decisions about management of adults with sore throat should incorporate both clinical estimates of the likelihood of a group A streptococcal infection and the priorities assigned to pertinent decision criteria by those affected by the decision. The results of this analysis provide an initial basis for determining the patient management implications of different priority judgments. Additional research is needed to more firmly establish the set of criteria that should be used to define quality management of adults presenting to primary care settings with sore throats and to assess the range of priorities patients, primary care practitioners and other interested parties assign to them. Until these results are available, we suggest that clinicians establish the likelihood of a Group A streptococcal infection using the Centor score and clinical setting, assess the relative priorities of avoid under-use versus over-use of antibiotics based on each patient's preferences and circumstances, and use the sensitivity analysis results presented in Table 2 to guide their patient management decisions.

## Competing interests

The author(s) declare that they have no competing interests.

## Authors' contributions

SS reviewed the literature, abstracted the information needed to make the outcome estimates and did the necessary calculations. He also helped prepare the manuscript. JGD helped generate the outcome estimates, did the AHP analyses, and helped prepare the manuscript. RMC conceived the study, participated in its design and completion, and helped to draft the manuscript. All authors read and approved the final manuscript.

**Table 1:** Data estimates used for the analysis

**Baseline assumptions**		**Reference**
Prevalence of Group A Streptococcal infection based on population prevalence of 10% and Centor Score Score = 1	3%	3
Score = 2	8 %	
Score = 3	19%	
Score = 4	41%	
Sensitivity of rapid streptococcal antigen test	84%	16
Specificity of rapid streptococcal antigen test	93%	16
Sensitivity of throat culture	78%	16
Specificity of throat culture	99%	16
Risk of penicillin rash	2%	17
Risk of anaphylaxis from oral penicillin	1/10,000	17
Risk of rheumatic fever if untreated	5/10,000	5, 15
Risk of rheumatic fever if treated	15/100,000	5, 15
Risk of peri-tonsillar abscess, if untreated	24/1,000	5, 15
Risk of peri-tonsillar abscess if treated	4/1,000	5, 15

**Table 2:** Results of one-way sensitivity analysis

**Group**	**Criterion**	**Baseline priority**	**Preferred option, baseline**	**Threshold value(s)**
1	Prevent complications	0.25	NO TEST, NO TREAT	NO TEST, NO TREAT < 0.406 < RAPID STREP & CULTURE or CULTURE
1	Avoid over- vs under use of antibiotics	0.5	NO TEST, NO TREAT	CULTURE or RAPID STREP & CULTURE < 0.375 < NO TEST, NO TREAT
2	Prevent complications	0.25	CULTURE	NO TEST, NO TREAT < 0.162 < CULTURE < 0.429 < RAPID STREP & CULTURE
2	Avoid side effects	0.25	CULTURE	CULTURE < 0.457 < NO TEST, NO TREAT
2	Optimal antibiotic use	0.25	CULTURE	RAPID STREP & CULTURE < 0.133 < CULTURE < 0.619 < NO TEST, NO TREAT
2	Avoid over- vs under use of antibiotics	0.5	CULTURE	RAPID STREP & CULTURE < 0.455 < CULTURE < 0.674 < NO TEST, NO TREAT
3	Prevent complications	0.25	RAPID STREP & CULTURE	NO TEST, NO TREAT < 0.15 < RAPID STREP & CULTURE
3	Avoid side effects	0.25	RAPID STREP & CULTURE	RAPID STREP & CULTURE < 0.354 < NO TEST, NO TREAT
3	Optimal antibiotic use	0.25	RAPID STREP & CULTURE	RAPID STREP & CULTURE < 0.389 < CULTURE
3	Avoid over- vs under use of antibiotics	0.5	RAPID STREP & CULTURE	EMPIRIC TREATMENT < 0.264 < RAPID STREP & CULTURE < 0.565 < NO TEST, NO TREAT
3	Minor penicillin side effect vs anaphylaxis	0.125	RAPID STREP & CULTURE	RAPID STREP & CULTURE < 0.44 < NO TEST, NO TREAT
4	Avoid treatment side effects	0.25	RAPID STREP & CULTURE	RAPID STREP & CULTURE < 0.545 < NO TEST, NO TREAT
4	Shorten duration of illness	0.25	RAPID STREP & CULTURE	RAPID STREP & CULTURE < 0.864 < CULTURE
4	Avoid over- vs under use of antibiotics	0.5	RAPID STREP & CULTURE	EMPIRIC TREATMENT < 0.264 < RAPID STREP & CULTURE < 0.737 < NO TEST, NO TREAT

## Pre-publication history

The pre-publication history for this paper can be accessed here:



## Supplementary Material

Additional File 1 summarizing the data used for the analysis. Click here for file

Additional File 2 summarizing the pairwise comparisons made among the expected outcomes of the alternative management strategies in the baseline analysis. Click here for file

Additional File 3 summarizing data used for the 20% Group A streptococcal prevalence population sensitivity analysis. Click here for file

Additional File 4 summarizing the pairwise comparisons made among the expected outcomes of the alternative management strategies in the 20% Group A streptococcal prevalence analysis. Click here for file

Additional File 5 illustrating the results of the one-way sensitivity analyses regarding the relative priorities of avoiding over-use versus under-use of antibiotics. Click here for file
