# Supplementary figures and images for: Optimal management of adults with pharyngitis – a multi-criteria decision analysis
Source: BMC Med Inform Decis Mak. 2006 Mar 13;6:14. doi: 10.1186/1472-6947-6-14 (PMC1431519; doi:10.1186/1472-6947-6-14)

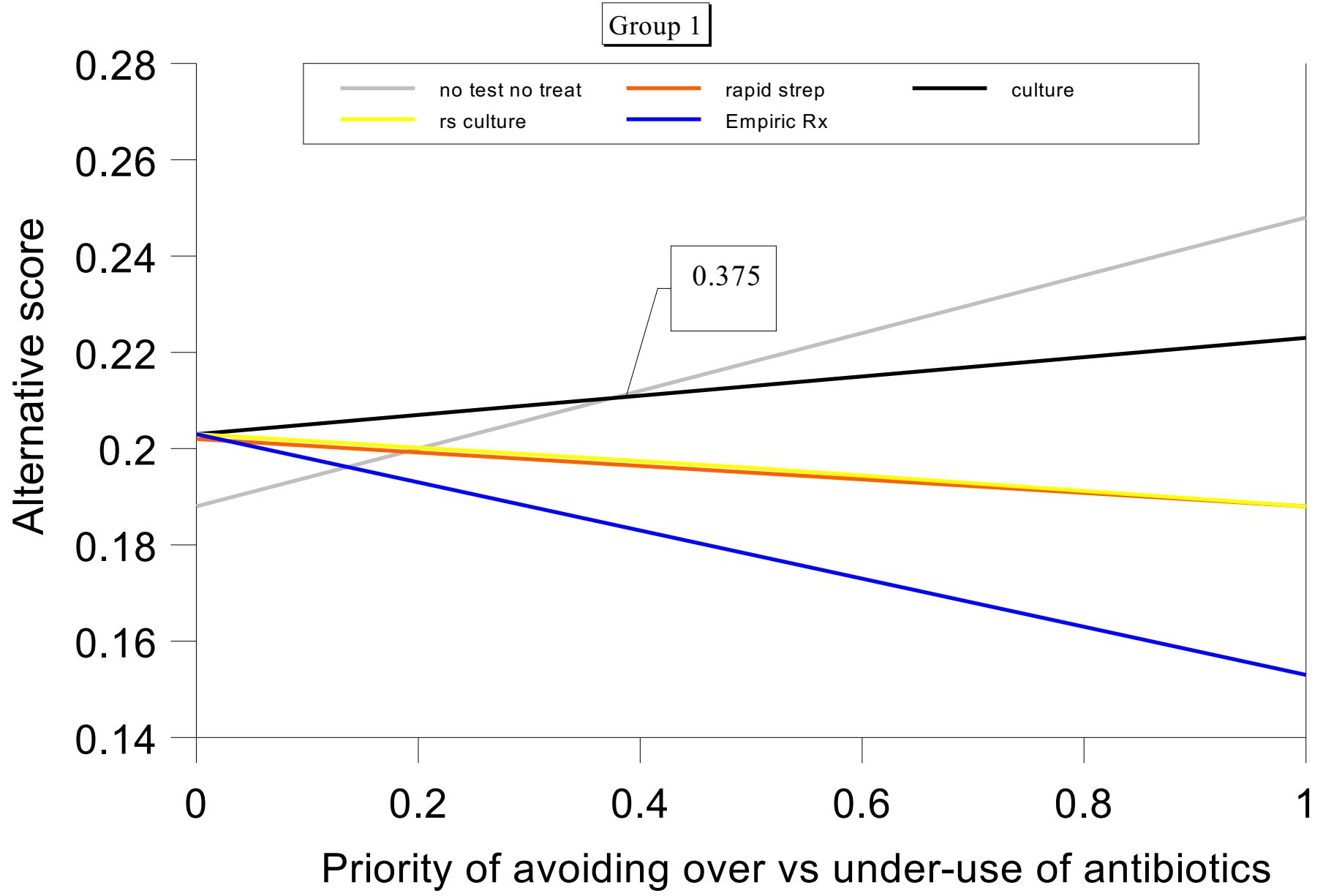

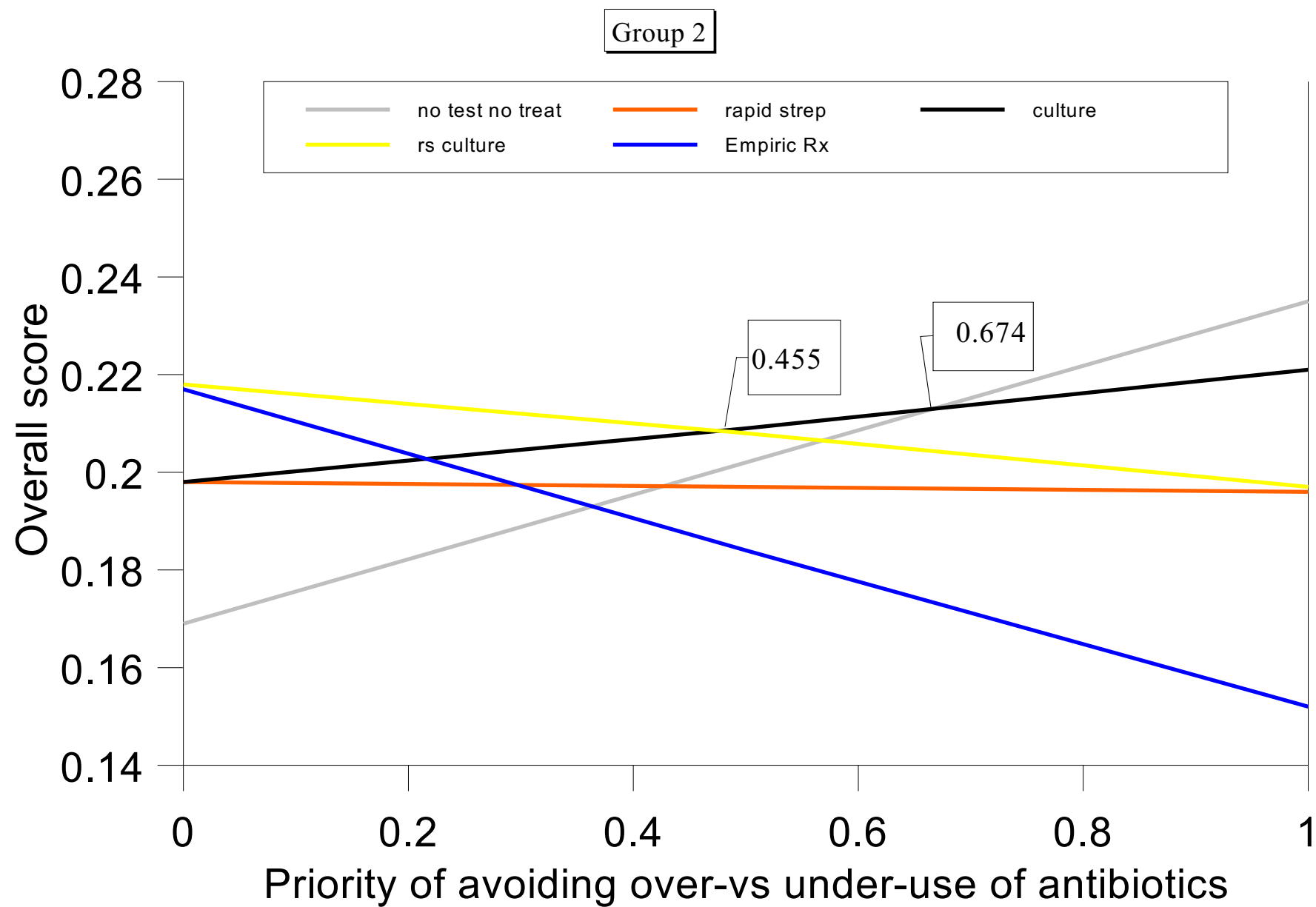

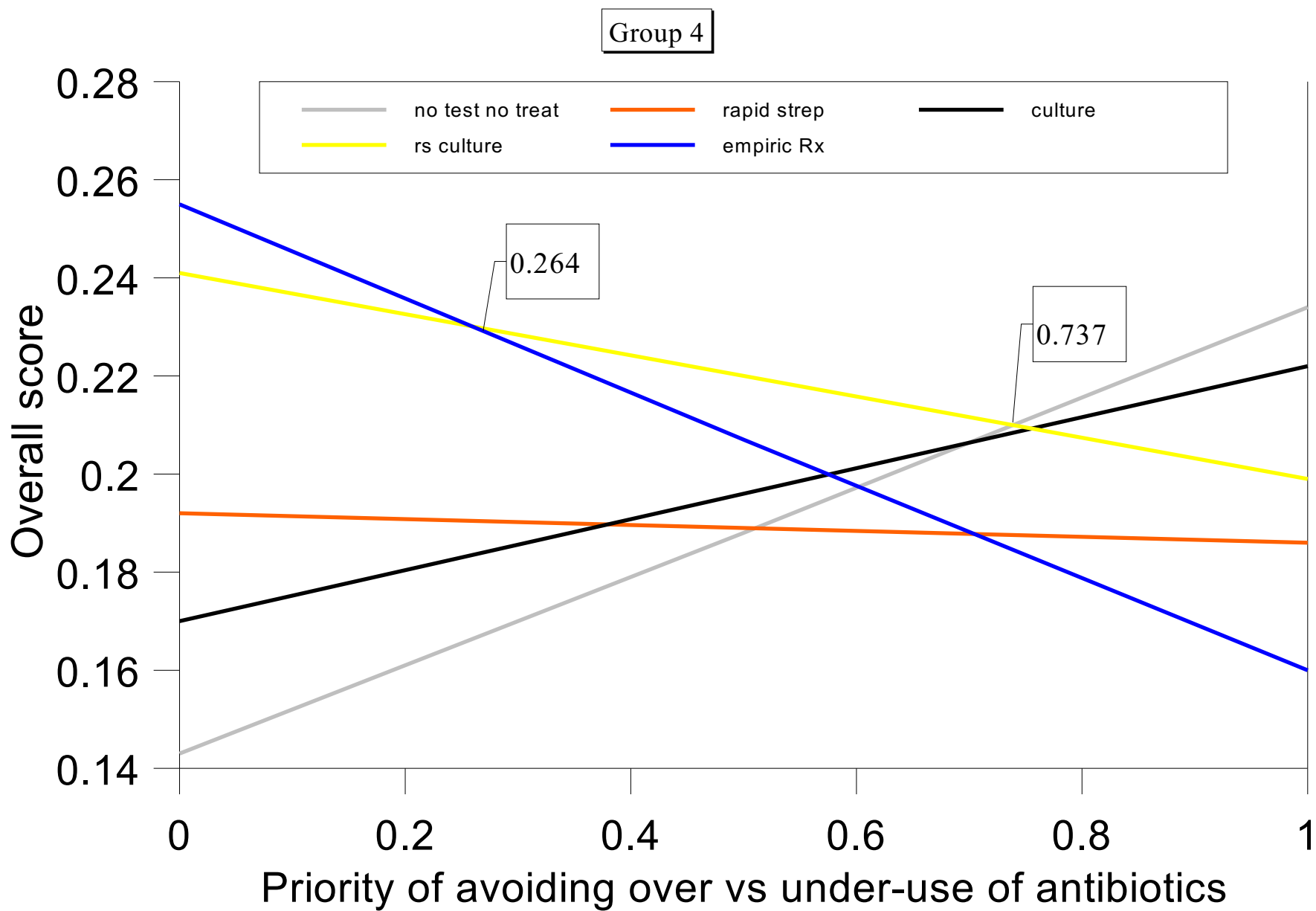

Supplement: Additional File 5 — illustrating the results of the one-way sensitivity analyses regarding the relative priorities of avoiding over-use versus under-use of antibiotics. [file 1472-6947-6-14-S5.pdf]
